# Editorial Comment: Evaluation of HIF-1α and VEGF-A expression in radiation-induced cystitis: A case-control study

**DOI:** 10.1590/S1677-5538.IBJU.2020.0054.1

**Published:** 2021-02-03

**Authors:** Rodolfo Borges dos Reis, Antonio Antunes Rodrigues, Rafael Neuppmann Feres, Valdair Francisco Muglia

**Affiliations:** 1 Universidade de São Paulo Faculdade de Medicina de Ribeirão Preto Departamento de Cirurgia e Anatomia Ribeirão Pretom SP Brasil Departamento de Cirurgia e Anatomia, Faculdade de Medicina de Ribeirão Preto, Universidade de São Paulo, Ribeirão Pretom, SP, Brasil; 2 Universidade de São Paulo Faculdade de Medicina de Ribeirão Preto Departamento de Imagens Médicas, Radioterapia e Oncohematologia Ribeirão Preto SP Brasil Departamento de Imagens Médicas, Radioterapia e Oncohematologia, Faculdade de Medicina de Ribeirão Preto, Universidade de São Paulo, Ribeirão Preto, SP, Brasil

## COMMENT

Radiation-induced late toxicity in normal tissue can impact quality of life of patients treated with radiotherapy. The authors studied the expression of the hypoxia-inducible factor-1 (HIF-1α) and its transcriptional target Vascular Endothelial Growth Factor A (VEGF-A) in patients with radiation cystitis due to therapy for locally advanced cervical cancer.

HIF is a cell mediator of adaptative responses in a hypoxic microenvironment regulating the expression of a cluster of genes. The high HIF-1 expression HAS been linked to tumor metastasis, angiogenesis, and tumor resistance therapy (1).

VEGF can induce growth of pre-existing (angiogenesis) or de novo vessels. VEGF-A is the most potent inducer of blood vessel growth and responsible to support tumor growth (2).

After bladder radiation, tissues with a slow turnover rate (vascular and connective) hold radiation damage at the time of treatment and the effects are not expressed until repeated cell division is undertaken, resulting in late toxicity.

Late radiation tissue damage can take several months to many years. Atrophy and fibrosis are the most frequent findings in irradiated tissues, probably caused by radiation-induced endothelial injury (3, 4).

Today there is no target therapy or marker to treat or prevent late toxicity of bladder radiation. A better understanding of all mechanisms involved is crucial to develop strategies to help patients.

In a case control-study, the authors studied the expression of HIF-1 α and VEGF-A on bladder core biopsies obtained from fourteen patients treated with chemoradiation therapy for locally advanced cervical cancer. The authors did not find any difference in expression of HIF-1α in irradiated bladder tissue vs controls (p=0.177); no difference in VEGF-A expression was detected also (p=0.250). Therefore their hypothesis of HIF induced neovascularization in radiation cystitis could not be confirmed (5).

Some limitations of the study were highlighted by the authors, the small number of patients and the quality of RNA extracted, certainly affected the results.

Radiation therapy aims to deliver high doses of radiation to target organ sparing the health tissues. Advances in radiation therapy (high-energy linear accelerators, conformal radiation therapy and intensity-modulated radiation therapy), has allowed to increase the dose, decreasing side effects (6). Radiation Cystitis is a result of many factors (7), clearly the type of radiation, the technique, and the total dose and fractions are important for planning treatment and can impact toxicity. The authors reported differences on radiotherapy equipment (cobalt, linear accelerator); technique (2D, 3D) and dose, furthermore eleven patients were treated also with brachytherapy and three were not. These differences may affect the urothelium differently.

HIF-1a expression has been linked to tissue regeneration and fibrosis in other tumors (8, 9), it plays a central role in activation of glycolytic enzymes providing energy for survival, allowing stem cells to keep their function, especially in adverse hypoxic conditions (10). Despite the unbalanced group of patients, the absence of high expression of HIF-1α and VEGF, instead of being associated with tissue hypoxemic conditions, may reflect the urothelial regenerative (angiogenesis) disability and late fibrosis, due to permanent tissue damage (DNA/cell damage) induced by radiation. In this scenario, “increased” vascularization, bleeding, and hemorrhagic cystitis may express the loss of the bladder mucosa barrier, due to regenerative incapability, rather than increased neovascularization. This finding is common on late stages of severe actinic cystitis. We congratulate the authors for the originality of the study.
